# Nephroprotective Effects of Caffeine, Vanillin, and Their Combination against Experimental AlCl_3_-Induced Renal Toxicity in Adult Male Wistar Rats

**DOI:** 10.1155/2023/6615863

**Published:** 2023-08-22

**Authors:** Olakunle Bamikole Afolabi, Oluwaseun Ruth Olasehinde, Oyindamola Adeniyi Olaoye, Kikelomo Folake Jaiyesimi, Ilobekemen Lisa Ekakitie, Omotade Ibidun Oloyede

**Affiliations:** ^1^Department of Chemical Sciences, Biochemistry Programme, College of Science, Afe Babalola University, P.M.B 5454, Ado-Ekiti, Ekiti State, Nigeria; ^2^Department of Medical Biochemistry, College of Medicine and Health Sciences, Afe Babalola University, P.M.B 5454, Ado-Ekiti, Ekiti State, Nigeria; ^3^Department of Biochemistry, Ekiti State University, P.M.B 5363, Ado-Ekiti, Ekiti State, Nigeria

## Abstract

Aluminum (Al) is known to be a nephrotoxic metal that can cause renal toxicity in both humans and animals. The use of functional foods has been reported to have significance in managing the toxic effects associated with such metals. This study aimed to assess the potential protective effects of caffeine, vanillin, and their combination in mitigating AlCl_3_-induced renal toxicity in adult male Wistar rats. A total of thirty (30) adult male Wistar rats weighing between 150 and 200 g were randomly divided into five groups, each consisting of six rats (*n* = 6). Group 1 served as the control, while the remaining treatment groups received a daily oral dose of 100 mg/kg AlCl_3_ for a duration of 21 days. In addition, groups 3–5 were coadministered 50 mg/kg body weight (bw) of caffeine, vanillin, and a combination (50/50 mg/kg bw) of both substances, respectively. In the results, AlCl_3_-treated showed a significant (*p* < 0.05) increase in serum biomarkers such as ALT, ALP, urea, and creatinine, and a significant (*p* < 0.05) decrease in serum total proteins (TPs). The renal tissue's antioxidant system, including SOD, CAT, GPx, and GSH, exhibited a significant (*p* < 0.05) reduction, accompanied by an elevated MDA level. However, the administration of caffeine, vanillin, and their combination resulted in a significant (*p* < 0.05) decrease in serum ALT, ALP, urea, and creatinine, and a significant (*p* < 0.05) increase in serum TP. Furthermore, following the treatment, there was a significant (*p* < 0.05) increase in renal SOD, CAT, GPx, and GSH levels, along with a reduction in the MDA level. In addition, the treatment for 21 days caused a significant (*p* < 0.05) reversal to the altered histomorphological architecture. These findings suggest that caffeine, vanillin, and their combination could potentially be an effective regimen in managing AlCl_3_-induced renal toxicity.

## 1. Introduction

Aluminum (Al) is a well-known environmental toxicant and a reactive waste product generated by industrial activities [1]. It is widely distributed and commonly found in various food products [2]. The potential risks associated with exposure to Al have raised significant concerns, highlighting the need for attention [3]. Epidemiological research has demonstrated that metal toxicants, including Al, play a crucial role in the development of chronic kidney toxicity [4]. The organ kidney is critical in maintaining homeostasis, detoxification, and excretion of both drugs and toxic metabolites; it appears to be a major target organ for toxicants [5]. The kidneys have the primary responsibility of eliminating the majority of Al compounds that enter the body through contaminated food, water, and environmental factors [6]. Consequently, the kidneys are the primary target for Al bioaccumulation. At elevated concentrations, Al accumulates in renal tissue and leads to nephrotoxicity [7].

Furthermore, numerous *in vitro* and *in vivo* experimental investigations have demonstrated that Al negatively influences cellular components, leading to the production of reactive oxygen species (ROS) such as superoxide anion, hydrogen peroxide, and hydroxyl radical [8]. In addition, Al disrupts mitochondrial function, resulting in the excessive generation of highly reactive free radicals/electrophiles [9]. These ROS can further damage cellular constituents such as lipids, proteins, and DNA, and interfere with the redox regulatory system [10]. However, recent studies suggest that neutralizing ROS is a crucial strategy in preventing or impeding the progression of pathological processes associated with Al exposure [11, 12]. The roles of antioxidants or other compounds that can scavenge or inhibit the formation of ROS have been reported in the reduction or neutralization of ROS [13]. By reducing the levels of ROS, this strategy restores the balance of oxidative stress and prevent further damage to cellular components or cellular dysfunction [14]. Recent reports have highlighted the significance of functional foods in managing pathological conditions associated with ROS by providing additional bioactive compounds and functional properties [15, 16]. Functional foods are known to contain natural antioxidants such as vitamins (e.g., vitamin C and E), minerals (e.g., selenium and zinc), phytochemicals (e.g., polyphenols and carotenoids), and other bioactive compounds [17]. These antioxidants directly interact with ROS, neutralizing them by donating electrons or hydrogen atoms and preventing their detrimental effects on cellular components [18].

Caffeine, a bioactive molecule found in coffee [19], has antioxidant and anti-inflammatory activities [20, 21]. It has been demonstrated to decrease lipid peroxidation, reduce ROS growth, and improve mitochondrial function in a variety of biological processes [22]. Similarly, vanillin (4-hydroxy-3-methoxybenzaldehyde), a primary aromatic component of vanilla [23], is found in a wide range of processed foods, medications, and perfumes [24]. According to research, vanillin has antioxidant, anti-inflammatory, anticarcinogenic, and mutagenic effects [25, 26]. However, there are little data on the possible therapeutic benefits of these drugs on aluminum-induced kidney damage. As a result, the focus of this study was to look into the nephroprotective potential of caffeine, vanillin, and their combination against aluminum chloride (AlCl_3_)-induced kidney damage in adult Wistar male rats.

## 2. Materials and Methods

### 2.1. Chemicals and Reagents Used

Chemicals and reagents such as AlCl_3_ and thiobarbituric acid (TBA) were procured from Sigma-Aldrich, Inc., (Saint Louis, MO, USA) while vanillin and caffeine were sourced from British Drug Houses (BDH) Chemical Ltd., Poole, England. Other biochemical assay kits such as aspartate aminotransferase (AST), alanine aminotransferase (ALT), total proteins (TPs), urea, and creatinine assay kits were procured from Randox Laboratory Ltd., Crumlin (Antrim, Northern Ireland, UK).

### 2.2. Sample Preparation

The stock solutions (concentrations 50 mg/kg each) of caffeine and vanillin were prepared in distilled water and kept in refrigerator (at −8°C) throughout the treatment period following a modified protocol of Akomolafe et al. [21].

### 2.3. Experimental Regimen

#### 2.3.1. Animal Treatment

A total of thirty adult male Wistar rats (*n* = 30) weighing between 150 and 200 g were obtained from the animal house at Afe Babalola University in Ado-Ekiti, Ekiti State, Nigeria. The animals were allowed to acclimatize under humane conditions for a period of 7 days at room temperature before the commencement of the study, which spanned 21 days (3 weeks). Throughout the duration of the study, experimental animals were housed at room temperature with a 12-hour light/dark cycle and provided with unrestricted access to pelletized animal feed (ABUAD LIVESTOCK FEED, produced by the Afe Babalola University Farm in Ekiti State, Nigeria; consisting of ingredients such as corn starch, soybean oil, dextrin starch (maltodextrin), casein, vitamins, and choline bitartrate) and water *ad libitum*. The animal treatment procedures conducted in this study adhered strictly to the Principles of Laboratory Animal Care [27].

### 2.4. Ethical Clearance

The use of experimental animals (rats) involved in this study was carried out with a strict compliance to the ethical guidelines for the best practice issued by the Ethical Clearance Committee (ECC) of Afe Babalola University with the applied ethical code: ABUAD/ACA/126.

### 2.5. Al Exposure Protocol

Exposure to Al for the induction of nephrotoxicity was carried out according to a modified method described by Kumar et al. [28]. Experimental rats received a daily oral dose of 100 mg/kg AlCl_3_ bw (dissolved in water) and 100 mg/kg caffeine, vanillin, and their combination for a period of twenty-one days.

### 2.6. Animal Grouping

Experimental animals were randomly divided into six treatment groups of 6 rats each (i.e., *n* = 6) as follows:

1. Group 1: normal control group
2. Group 2: untreated AlCl_3_-treated control group
3. Group 3: AlCl_3_-treated + oral gavage of 50 mg/kg vanillin
4. Group 4: AlCl_3_-treated + oral gavage of 50 mg/kg caffeine
5. Group 5: AlCl_3_-treated + oral gavage of vanillin + caffeine (50 + 50 mg/kg bw)

### 2.7. Collection and Preparation of Tissues

#### 2.7.1. Preparation of Blood Sample

Experimental animals were euthanized by mild exposure to diethyl ether, after overnight fasting, following the withdrawal of feed and water. Blood samples were immediately collected by direct heart puncture into plain sample bottles. Blood samples were subsequently centrifuged at 1800 ×g at 27°C for 10 min to obtain the sera used for the various biochemical analyses.

#### 2.7.2. Preparation of Tissue Homogenate

Kidney tissues were subsequently dissected and rinsed in 0.1 M tris buffer (pH 7.4), blotted with filter paper, and placed on ice. Each sample was weighed and subsequently homogenized in 0.1 M tris buffer (1 : 5 w/v). The homogenate was centrifuged at 3000 rpm for 10 min with the pellet discarded and the supernatant kept in refrigerator for various biochemical assays.

#### 2.7.3. Biochemical Assays

Determination of serum biomarkers such as aspartate aminotransferase (AST) and alanine aminotransferase (ALT) activity assays were carried out using the method described by Reitman and Frankel [29], while serum total proteins (TPs), urea, and creatinine were determined according to the user manuals (Randox reagent kits). Enzymatic antioxidant parameters of the kidney tissues such as superoxide dismutase (SOD), catalase (CAT), glutathione peroxidase (GPx), and nonenzymatic antioxidant parameter such as reduced glutathione (GSH) and malondialdehyde (MDA) contents produced were determined using the method described by Oloyede et al. [30].

#### 2.7.4. Histological Study

Histological alterations in the renal tissue samples were analyzed according to the method used by Oloyede et al. [30]. Samples were isolated from the animals after sacrifice and preserved in formalin (10%) solution. Thereafter, tissues were embedded in paraffin. A microtome was used to collect micrometer-thick paraffin sections, and tissues were then stained with hematoxylin and eosin (H&E). Examination of possible changes was carried out under a light compound microscope.

#### 2.7.5. Statistical Analyses

The collected data were subjected to statistical analysis using the one-way analysis of variance (ANOVA) test in the SPSS software (Evaluation Version 16.0, SPSS Inc., Chicago, IL, USA). Post hoc comparisons were conducted using the Duncan multiple range test when necessary. Graphical representations of the data were generated using the GraphPad Prism 9.0 program (GraphPad Software, San Diego, CA, USA). Statistical significance was determined at a *p* value of less than 0.05, and the results were presented as the mean ± standard error of the mean (SEM) from 6 trial determinations (*n* = 6).

## 3. Results

### 3.1. General Observation

At the end of the experimental window, animals in AlCl_3_-induced nephrotoxicity group showed slight weakness, reduction in weight and feed consumption as well as furs loss when compared to the normal rats (control), vanillin-, and caffeine-treated groups. It should also be noted that no mortality was recorded in this study based on the different treatments among either the AlCl_3_-induced nephrotoxicity group or other groups.

#### 3.1.1. Effects of Caffeine, Vanillin, and Their Combination on Serum Biomarkers and Total Proteins during AlCl_3_-Induced Renal Toxicity in Adult Male Rats

**Figure 1:**
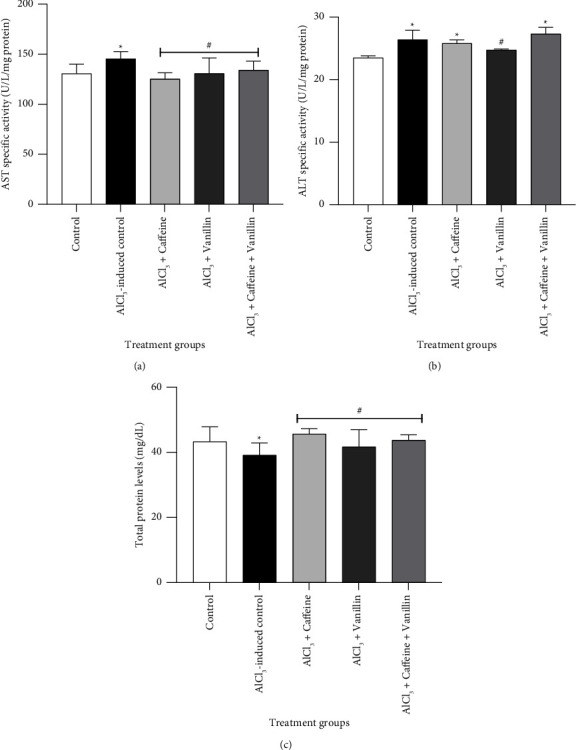
Effects of caffeine, vanillin, and their combination on (a) AST-specific activity, (b) ALT-specific activity, and (c) serum total proteins in AlCl_3_-induced renal toxicity in adult male rats. Results are expressed as mean ± SEM of six determinations (*n* = 6). Note: ^*∗*^ indicates *p* < 0.05 compared to normal control; ^#^*p* < 0.05 compared to AlCl_3_-induced nephrotoxicity.

Figures 1(a)–1(c) illustrates the effects of caffeine, vanillin, and their combination on serum-specific biomarkers and TP levels during AlCl_3_-induced renal toxicity in adult male Wistar rats. The results demonstrate a significant (*p* < 0.05) increase in AST and ALT activities, along with a significant (*p* < 0.05) reduction in TP levels of the AlCl_3_-induced nephrotoxicity compared to the normal control. However, treatment with 50 mg/kg caffeine, vanillin, and their combination resulted in a significant (*p* < 0.05) reduction in the activities of serum AST and ALT, accompanied by a significant (*p* < 0.05) increase in TP levels, compared to both the normal control and the AlCl_3_-induced nephrotoxicity group.

#### 3.1.2. Effects of Caffeine, Vanillin, and Their Combination on Renal Creatinine and Urea Levels in AlCl_3_-Induced Renal Toxicity in Adult Male Rats

**Figure 2:**
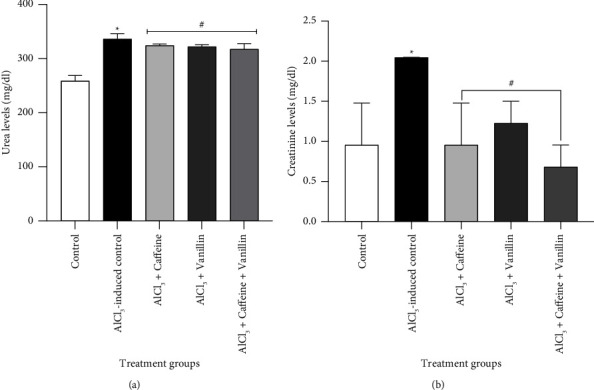
Effects of caffeine, vanillin, and their combination on (a) urea levels and (b) creatinine levels in AlCl_3_-induced renal toxicity in adult male rats. Results are expressed as mean ± SEM of six determinations (*n* = 6). Note: ^*∗*^indicates *p* < 0.05 versus normal control; ^#^*p* < 0.05 versus untreated AlCl_3_-induced nephrotoxicity.

Figures 2(a) and 2(b) depict the effect of caffeine, vanillin, and their combination on renal function markers during AlCl_3_-induced renal toxicity in adult male Wistar rats. The results demonstrate a significant (*p* < 0.05) increase in urea and creatinine levels in the AlCl_3_-induced nephrotoxicity group compared to the normal control. However, treatment with 50 mg/kg caffeine, vanillin, and their combination resulted in a significant (*p* < 0.05) reduction in urea and creatinine levels compared to both the normal control and the AlCl_3_-induced nephrotoxicity group.

#### 3.1.3. Effects of Caffeine, Vanillin, and Their Combination on Tissue Antioxidant Parameters in AlCl_3_-Induced Renal Toxicity in Adult Male Rats

**Figure 3:**
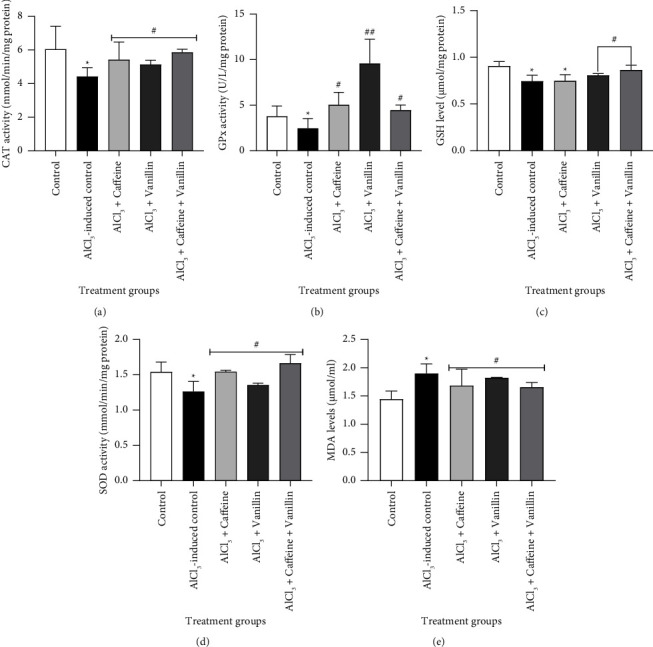
Effects of caffeine, vanillin, and their combination on renal tissue (a) CAT activity, (b) GPx activity, (c) GSH level, (d) SOD activity, and (e) MDA levels in AlCl_3_-induced toxicity in adult male rats. Results are expressed as mean ± SEM of six trials (*n* = 6). ^*∗*^indicates a significant difference at *p* < 0.05 versus normal control; ^#^*p* < 0.05 versus untreated AlCl_3_-induced nephrotoxicity; ^##^*p* > 0.05 versus untreated AlCl_3_-induced nephrotoxicity and normal control.

Figures 3(a)–3(e) represents the effects of caffeine, vanillin, and their combination on renal tissue antioxidant parameters against AlCl_3_-induced toxicity in adult male rats. As shown in the results, there was a significant (*p* < 0.05) decrease in SOD, CAT, and GPx activities as well as a reduction in GSH levels of AlCl_3_-induced nephrotoxicity group with a significant (*p* < 0.05) increase in MDA levels of the AlCl_3_-induced nephrotoxicity compared to normal. However, administration of 50 mg/kg caffeine, vanillin, and their combination caused a significant (*p* < 0.05) increase in the activities of SOD, CAT, and GPx activities as well as GSH levels with a significant (*p* < 0.05) increase in MDA level when compared to AlCl_3_-induced nephrotoxicity.

#### 3.1.4. Effects of Caffeine, Vanillin, and Their Combination on Histological Architecture of AlCl_3_-Induced Renal Toxicity in Adult Male Rats

**Figure 4:**
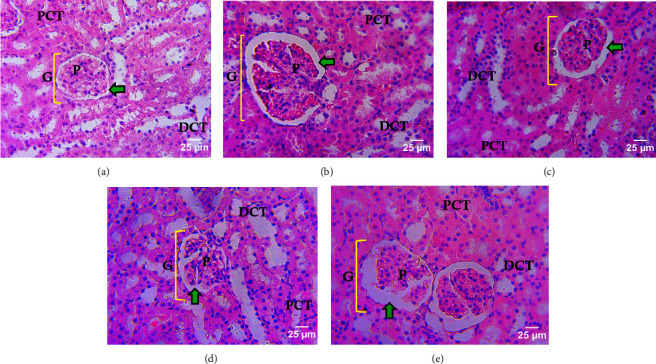
Photomicrographs of hematoxylin-eosin-stained renal tissues (x800) of AlCl_3_-induced toxicity in male Wistar rat. G, glomerulus; PCT, proximal convoluted tubules; DCT, distal convoluted tubules; green arrow, urinary space; (a) normal control; (b) untreated AlCl_3_-induced nephrotoxicity; (c) AlCl_3_-induced + 50 mg/kg vanillin; (d) AlCl_3_-induced + 50 mg/kg caffeine; (e) AlCl_3_-induced + vanillin/caffeine (50/50 mg/kg).

Figures 4(a)–4(e) shows the effects of caffeine, vanillin, and their combination on histological status of AlCl_3_-induced renal toxicity in adult male rats. As indicated in Figure 1(a), AlCl_3_-induced nephrotoxicity showed enlargement of the urinary space, DCT, and PCT compared to normal control with a normal architecture of the glomerulus showing moderate loss of PCT and DCT cells. However, group administered with 50 mg/kg bw vanillin revealed a slightly enlarged urinary space, loss of cuboidal cells in some DCT and PCT with 50 mg/kg bw caffeine-treated group revealed normal architecture of the glomerulus, moderate degeneration of the PCT and DCT cells, while the group treated with combination of vanillin and caffeine revealed enlargement of the urinary space and degeneration of the glomerular cells.

## 4. Discussion

Nephrotoxicity is a condition in which the kidneys detoxification and excretion functions are impaired as a result of toxicants or drug-induced damage [31]. Al is a well-known toxicant that can accumulate in various tissues of mammals, including the kidneys, liver, heart, blood, bones, and brain [32]. Among these tissues, the kidneys are particularly vulnerable to Al exposure, as they play a crucial role in preventing its bioaccumulation through excretion mechanisms [33]. The objective of this study was to evaluate the nephroprotective effects of caffeine, vanillin, and their combination in preventing and/or reducing kidney damage induced by AlCl_3_ in adult male rats. This was achieved by assessing various biochemical parameters related to renal function in an experimental model of induced renal toxicity.

According to a study reported by Ghlissi et al. [34], parameters such as ALT and AST are considered indicators of nephrotoxicity. In addition, Abadi et al. [35] found that elevated serum levels of transaminases (ALT and AST) are often released from the cell cytosol due to cellular necrosis, indicating cytotoxicity. When an organ experiences cellular degeneration or damage, the plasma levels of these markers tend to increase [36]. The observed increase in serum activities of AST and ALT, as seen in Figures 1(a) and 1(b), can be attributed to the cytotoxic effect of Al on the kidney membrane permeability [37], which could lead to the leakage of these enzymes from the cytosol into bloodstream, along with disruptions or dysfunction in their activation [38]. Conversely, a slight decrease in total protein levels observed in AlCl_3_-induced rats may be due to an increase in intracellular Al concentration, which has been shown to reduce protein synthesis [39], aligning with the findings of Nawel et al. [40]. However, the administration of caffeine, vanillin, and their combination resulted in decreased activities of ALT and AST, as well as an increase in plasma protein concentration (Figure 1). This suggests a nephroprotective effect of caffeine and vanillin against AlCl_3_-induced nephrotoxicity, possibly through the preservation of renal membrane permeability or their ability to reduce intracellular Al concentration. This effect is further supported by the significant improvement observed in the renal histological architecture, as indicated in Figure 4.

Serum creatinine and urea concentrations are widely utilized as indicators of injury in studies involving nephrotoxicity or renal dysfunction [41]. Creatinine is a metabolic byproduct generated in muscles from high-energy storage compounds such as creatine phosphate [42]. It is eliminated from the bloodstream via glomerular filtration in the kidneys and subsequently excreted in urine with minimal reabsorption by the renal tubules [43]. Conversely, urea is a nitrogenous waste product that undergoes filtration by the glomerulus, reabsorption by the renal tubules, and excretion in urine [44]. The plasma concentration of urea is dependent on the rate of excretion by the kidneys [45]. The elevation of creatinine and urea levels observed in the AlCl_3_-induced nephrotoxicity (Figure 2) may indicate severe renal injury associated with Al exposure, leading to a reduction in glomerular filtration rate and impaired clearance of serum creatinine, as well as abnormal retention of urea [46]. However, treatment with caffeine, vanillin, and their combination resulted in a significant reduction in plasma urea and creatinine levels, indicating potential renoprotective effects against Al-induced kidney toxicity. This protective effect could be attributed to their ability to inhibit arginase induction, which is involved in urea production, or to their ability to improve glomerular filtration rate, thereby facilitating proper clearance of creatinine and urea [47].

Intracellular antioxidant systems play a critical role in protecting against nephrotoxicity induced by heavy metals [48]. Under normal physiological conditions, the balance between the generation and elimination of reactive oxygen species (ROS) maintains cellular function, while disturbances in redox homeostasis can lead to oxidative stress [49]. Al has been shown to disrupt the body's intrinsic antioxidant defense mechanisms by promoting lipid peroxidation (LPO) through the mediation of Fe^2+^ ions [33]. This interference with cellular redox state inhibits enzymes such as superoxide dismutase (SOD), which converts superoxide radicals into H_2_O_2_ and O_2_, as well as glutathione peroxidase and catalase (CAT), which detoxify H_2_O_2_ to H_2_O at the expense of reduced glutathione (GSH) [50]. This disruption results in an elevated production of ROS and contributes majorly to the occurrence cellular oxidative damage [51–53]. A decrease in CAT, SOD, GPx, and GSH as indicated in this report (Figures 3(a)–3(d)) could possibly reflect Al-mediated renal oxidative damage prompting an increased MDA, which is a biomarker of LPO [46]. This finding corroborates the report of Liu et al. [54]. However, administration of caffeine, vanillin, and their combination caused an increase in the levels of these proteins with a concomitant decrease of MDA possibly by mitigating Al-induced ROS proliferation, thereby promoting protection in the kidney against nephrotoxic effect of Al [55].

Furthermore, a normal histoarchitecture of the kidney plays a key role in its excretory and homeostatic functions [56]. In our study, a variety of structural alterations were noticed in the renal histology of the AlCl_3_-induced groups, an effect that probably have reflected Al-induced oxidative assault and derangement of both the proximal and distal convoluted tubules (Figure 4) [57, 58]. In this study, treatment with caffeine and vanillin significantly improved and restored renal histological status such as normal glomerulus, moderate degeneration of the proximal, and distal convoluted tubules with a decrease in urinary space. However, the group treated with the combination of vanillin and caffeine revealed an increase in urinary space and mild degeneration of the glomerular cells. This effect probably indicated that the combination had no significant effect improving the urinary space in nephrotoxicity.

## 5. Conclusion

This study evaluated nephroprotective effects of caffeine, vanillin, and their combination in AlCl_3_-induced nephrotoxicity. Our finding revealed that all these compounds demonstrated significant improvement in biochemical parameters and restoration of histoarchitectural status, which are crucial for normal excretory and homeostatic functions of the kidneys. Therefore, caffeine, vanillin, and their combination could be effective nephroprotective regimens in the management of AlCl_3_-related renal toxicity.

## Data Availability

No underlying data were collected or produced in this study.
